# An Ensemble Model for Fundus Images to Aid in Age-Related Macular Degeneration Grading

**DOI:** 10.3390/diagnostics15202644

**Published:** 2025-10-20

**Authors:** Roberto Romero-Oraá, María Herrero-Tudela, María Isabel López, Roberto Hornero, Pere Romero-Aroca, María García

**Affiliations:** 1 Biomedical Engineering Group, University of Valladolid, 47011 Valladolid, Spain; maria.herrero.tudela@uva.es (M.H.-T.); roberto.hornero@uva.es (R.H.); maria.garcia.gadanon@uva.es (M.G.); 2 Centro de Investigación Biomédica en Red en Bioingeniería, Biomateriales y Nanomedicina (CIBER-BBN), 47011 Valladolid, Spain; 3 Servei d’Oftalmologia, Hospital Universitari Sant Joan de Reus, Institut d’Investigaci’o Sanitària Pere Virgili (IISPV), Universitat Rovira i Virgili, 43204 Tarragona, Spain; pedro.romero@salutsantjoan.cat

**Keywords:** age-related macular degeneration, medical diagnosis, fundus images, deep learning, ensemble model

## Abstract

**Background:** Age-related macular degeneration (AMD) is a leading cause of visual impairment in the elderly population. Periodic examinations through fundus image analysis are paramount for early diagnosis and adequate treatment. Automatic artificial intelligence algorithms have proven useful for AMD grading, with the ensemble strategies recently gaining special attention. **Methods:** This study presents an ensemble model that combines 2 individual models of a different nature. The first model was based on the ResNetRS architecture and supervised learning. The second model, known as RETFound, was based on a visual transformer architecture and self-supervised learning. **Results:** Our experiments were conducted using 149,819 fundus images from the Age-Related Eye Disease Study (AREDS) public dataset. An additional private dataset of 1679 images was used to validate our approach. The results on AREDS achieved a quadratic weighted kappa of 0.7364 and an accuracy of 66.03%, which outperforms the previous methods in the literature. **Conclusions:** The ensemble strategy presented in this study could be useful for the screening of AMD in a clinical setting. Consequently, eye care for AMD patients would be improved while clinical costs and workload would be reduced.

## 1. Introduction

Age-related macular degeneration (AMD) is a leading cause of severe visual impairment in people over 50 years of age [1]. It affects 196 million people worldwide and this number is estimated to reach 288 million by 2040 [1]. Adequate treatment requires early diagnosis. However, the disease is initially asymptomatic, which requires periodic ophthalmologic examinations of the retinal fundus [2]. These examinations are mainly based on the analysis of fundus images, since they are the most cost-effective imaging modality [3]. Fundus imaging allows detailed visualization of the retina’s macular region, where drusen deposits, pigmentary changes, and neovascularization—the hallmarks of AMD—typically appear. The affordability and ease of acquisition of these images make them an optimal screening tool for population-wide monitoring of AMD progression.

Given the high prevalence of AMD and the lack of specialists trained to diagnose it, automatic artificial intelligence algorithms have proven useful for the screening of the disease [3]. Such automated systems are particularly valuable in rural or under-resourced regions, where access to expert ophthalmological evaluation is limited. They can be integrated into teleophthalmology platforms, allowing retinal images acquired in primary care centers to be remotely graded by AI-based systems. These algorithms can also support large-scale screening campaigns, reduce diagnostic delays, and help close healthcare gaps by ensuring that patients with early-stage AMD are identified and referred for timely management. In this way, automated AMD grading contributes to improving access to eye care and reducing preventable vision loss on a population scale.

Automatic retinal image analysis based on convolutional neural networks (CNNs) and supervised learning (SL) has been widely explored in the recent literature. OverFeat [4,5], Alexnet [6], VGG-16 [7], ResNet50 [7,8], ResNet101 [3] and EfficientNetB4 [3] architectures have been individually explored. Tan et al. [9] developed a 14-layer ad hoc architecture as a fast, portable solution. A previous comparison among individual CNN architectures showed that the ResNetRS [10] architecture achieved the highest performance for AMD detection [11]. However, ensemble strategies are gaining special attention for AMD grading. For example, Grassman et al. [12] combined AlexNet, VGG16, GoogLeNet, Inception-v3, ResNet101 and InceptionResNet-v2 to build a 13-class grading system. Govindaiah et al. [13] combined Inception-ResNet-V2 and Xception architectures to classify AMD into 4 levels. The DeepSeeNet model consists of 3 CNNs based on the Inception-v3 architecture and can classify 6 degrees of AMD [14]. It is also worth noting that the 5 best-ranked teams in the ADAM challenge proposed ensemble CNN strategies [3]. These ensemble strategies leverage the strengths of multiple models to provide a more robust and generalizable classification, which is especially important in a task with subtle inter-class differences, such as AMD grading. In real-world scenarios, ensemble models often outperform single-model approaches by mitigating biases, reducing variance, and improving overall confidence in predictions. This is particularly relevant in clinical diagnostics, where uncertainty may lead to inappropriate management.

Despite the success of CNNs, the emergence of the vision transformer (ViT) architecture has also revolutionized image classification models [15]. ViTs have demonstrated an exceptional ability to capture long-range dependencies and global context in images by replacing traditional convolutional layers with attention-based mechanisms. In this context, a foundation model for retinal images has recently been released under the name of RETFound [16]. This model is based on the ViT architecture and was trained with self-supervised learning (SSL) using unlabeled fundus images. RETFound has proven effective for the diagnosis of various retinal conditions, including diabetic retinopathy and glaucoma, via fine-tuning on task-specific retinal image datasets. In the original work, RETFound achieved area under the ROC curves (AUROCs) of approximately 0.90–0.97 in ocular disease classification tasks, outperforming standard supervised networks pretrained on ImageNet. Its pretraining strategy allows it to extract meaningful features from massive amounts of data without requiring manual labeling, which is particularly useful in medical imaging scenarios where expert annotation is expensive and time-consuming. Moreover, RETFound represents a paradigm shift in the development of medical artificial intelligence (AI): the use of large-scale self-supervised pretraining tailored to domain-specific data, followed by fine-tuning for specific diagnostic tasks.

The main motivation of this work stems from the need for automated AMD grading systems that are both accurate and generalizable across diverse imaging conditions and acquisition devices. While existing CNN-based methods have achieved competitive performance, they often struggle to generalize due to limited representational diversity. Conversely, transformer-based models such as RETFound capture broader contextual information but may lack sensitivity to fine-grained retinal features. By integrating both architectures through an ensemble strategy, we aim to leverage their complementary strengths—achieving more robust and clinically reliable predictions. This hybrid approach not only improves performance metrics over individual models but also enhances interpretability and transferability to independent datasets, addressing key limitations identified in prior studies.

Preliminary experiments with individual architectures confirmed that ResNetRS outperformed other CNNs in accuracy, while RETFound achieved competitive generalization through its self-supervised pretraining. Given the individual success of both mentioned approaches (CNN-SL and ViT-SSL), we hypothesize that their ensemble would benefit from a higher performance. This hypothesis is grounded in the expectation that the two types of models, trained with different learning paradigms and architectural principles, will offer diverse yet complementary feature representations. Our objective was to build an ensemble model by mixing a deep CNN and RETFound for the automatic grading of AMD using fundus images. The great difference in the architectures (CNN vs. ViT) and the type of learning (SL vs. SSL) suggests that the individual models can provide complementary features for the classification task. Combining such heterogeneous models not only enhances performance but may also improve robustness across varied datasets and imaging conditions. To the best of our knowledge, these types of models have never been ensembled for retinal analysis. This work, therefore, contributes a novel hybrid strategy to the field of automated ophthalmologic diagnosis. By investigating the synergy between CNN-based and transformer-based feature extraction, we aim to bridge the gap between legacy vision models and emerging large-scale AI in medical imaging. The main contributions of this work can be summarized as follows:

(1) We propose a novel ensemble model that combines a CNN architecture (ResNetRS) trained with SL and a transformer-based foundation model (RETFound) trained with SSL for AMD grading using fundus images.
(2) To the best of our knowledge, this is the first study to combine RETFound with a CNN model in an ensemble framework, demonstrating their complementary feature representations.
(3) The proposed approach was evaluated using the largest publicly available AMD dataset (AREDS) and externally validated on an independent clinical dataset, showing improved generalization and robustness.
(4) The results outperform previously published methods in terms of quadratic weighted kappa (QWK), confirming the effectiveness of the ensemble strategy for ordinal AMD classification.
(5) The study contributes a generalizable framework that can be extended to other retinal diseases and supports the development of clinically reliable AI-assisted diagnostic tools.

## 2. Materials and Methods

### 2.1. Datasets

The development and evaluation of the proposed method was carried out using the Age-Related Eye Disease Study (AREDS) public dataset [17]. An additional validation was conducted afterwards using a private dataset of fundus images. This dual-dataset strategy allows us to measure both the internal performance and external generalization of the proposed model.

The AREDS dataset was provided by the National Eye Institute as a long-term multi-center, prospective study [17]. It contains 172,800 fundus images from 4757 participants aged between 55 and 80 years old. However, we only used the 149,819 images that were centered in the macula. The AREDS dataset was graded into 6 levels according to the simplified AREDS severity scale: grade 1 (No AMD), grade 2 (early AMD), grade 3a (intermediate AMD a), 3b (intermediate AMD b), 4a (late AMD a), and 4b (late AMD b). However, classes 3a and 3b were merged into a single “Intermediate AMD” category, and classes 4a and 4b into a single “Late AMD” category. This grouping follows previous literature [13] and reflects clinically meaningful stages of disease progression while reducing class imbalance. Preliminary experiments using the original six-class AREDS configuration (1, 2, 3a, 3b, 4a, 4b) revealed that the increased class imbalance led to reduced model stability and a decrease in model performance. Consequently, we adopted the four-class grouping to ensure more robust and reliable results while maintaining clinical relevance. The resulting four categories were: (1) No AMD, (2) Early AMD, (3) Intermediate AMD, and (4) Late AMD. This 4-class simplification reduces complexity while maintaining clinical relevance and diagnostic utility. The distribution of images per AMD category in the AREDS dataset was as follows: 36,867 for No AMD, 32,509 for Early AMD, 51,809 for Intermediate AMD, and 28,634 for Late AMD. Our experiments followed a holdout strategy where the AREDS dataset was split into training, validation and test sets with an 80:10:10 ratio (119,805, 15,011 and 15,003 images, respectively). We ensured that all the images from a patient were included in the same set. This patient-level splitting strategy avoids data leakage and better reflects real-world clinical settings.

The private dataset used for validation purposes was provided by the Hospital Universitario Sant Joan de Reus (HUSJR). It consists of 1679 fundus images captured with a DRI OCT Triton camera. This dataset was graded into 4 AMD categories using the same criteria as for the AREDS dataset. The distribution of images per AMD category in the private dataset was as follows: 925 for No AMD, 271 for Early AMD, 228 for Intermediate AMD, and 255 for Late AMD. It is important to mention that the validation procedure required fine-tuning the proposed model using a subset of the private dataset due to the great differences between AREDS and the private database. The AREDS images were collected between 1992 and 2004 using early-generation fundus cameras, resulting in an average resolution of approximately 640 × 560 pixels and noticeably lower color contrast and illumination uniformity compared to modern imaging systems. In contrast, the private dataset consisted of higher-resolution images (average 3008 × 2000 pixels) acquired with recent digital cameras, showing improved sharpness and contrast-to-noise ratios. Such discrepancies in imaging conditions between datasets are typical in multi-center medical imaging research and must be carefully managed. For the fine-tuning task, we split the private dataset into training, validation and test sets with a 50:25:25 ratio (839, 420, and 420 images, respectively). This additional fine-tuning ensured that our model could adapt to different domains and imaging characteristics.

### 2.2. Overview

**Figure 1:**
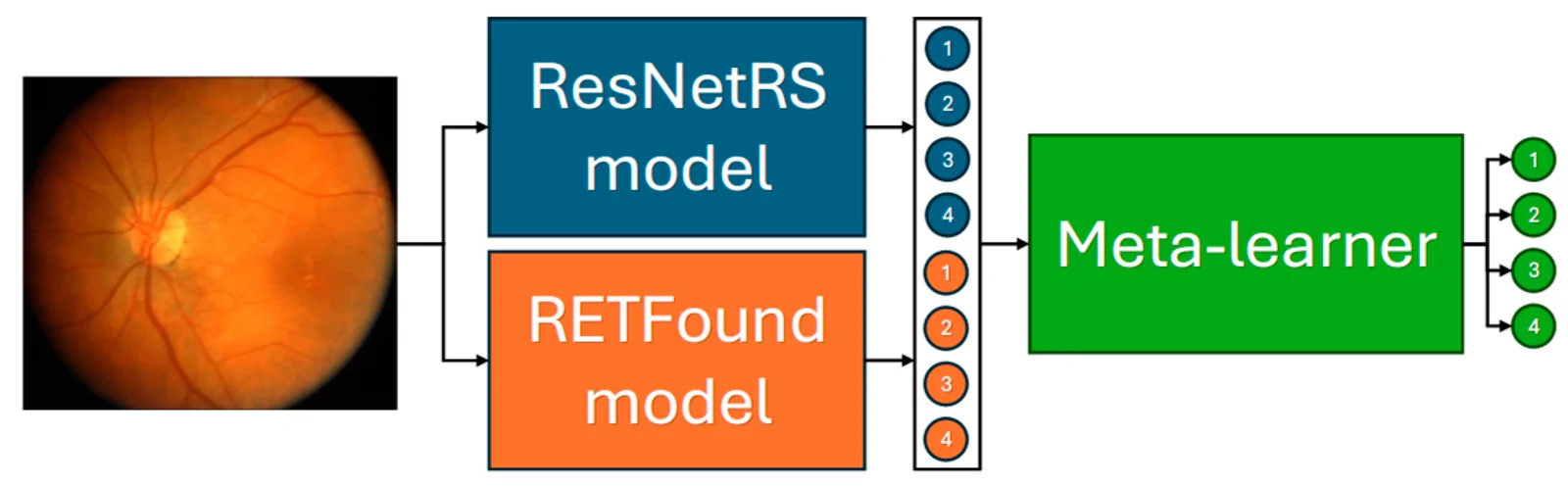
Overview of the ensemble model. The input image is processed by the individual models. Their outputs are then concatenated to be the input of the meta-learner. Numbers 1–4 represent the four AMD categories: (1) No AMD, (2) Early AMD, (3) Intermediate AMD, and (4) Late AMD.

The input images were first adapted to the subsequent processing through a preprocessing stage. Then, we built 2 individual models that were independently trained to grade AMD into 4 degrees. The first model was based on the ResNetRS architecture [10] while the second model was based on RETFound [16]. Finally, we developed an ensemble model which takes the outputs of the individual models as the input. Figure 1 shows a scheme of the final model. This ensemble approach follows a late fusion strategy, where model predictions are combined at the decision level to produce the final classification.

### 2.3. Preprocessing

First, the retinal region was automatically detected by selecting the largest area that exceeded a minimum intensity threshold (empirically obtained). The images were cropped to remove the surrounding black background to ensure that only the retinal area was retained. Before further processing, all images were resized to a resolution of 224 × 224 pixels, as in previous studies [13,16]. In addition, pixel values were normalized to the range [0, 1]. This standardization allows the models to learn more effectively and avoids issues related to scale variance or dynamic range disparities.

### 2.4. Data Augmentation

We applied online data augmentation to generate new synthetic images on the fly in every training epoch. The generated images were obtained from the training set by means of simple transformations: rotations (±50°), shifts (±7%), flips and scaling (±10%). This way, we increased the diversity of the training set to improve generalization [12]. Online augmentation also acts as a form of regularization, reducing overfitting and improving the robustness of the model.

### 2.5. ResNetRS Model

We used the ResNetRS architecture as the backbone of our CNN model [10]. This architecture, which can be seen as an improved version of the canonical ResNet, introduces a new training methodology and two scaling strategies. At the end of this architecture, we added a global average pooling layer and 3 dense layers of 1024, 512 and 4 neurons, respectively. The first 2 dense layers had a ReLU activation function and the last one a softmax. In between the dense layers, we applied dropout with a factor of 0.4 to help prevent overfitting [18].

To accelerate convergence and facilitate the learning task, the initial weights of our backbone were pretrained on the ImageNet database [19]. Then, we trained our model for 100 epochs with early stopping using a batch size of 16 images and a linear warmup with cosine annealing learning rate schedule. We used the weighted categorical cross entropy as the loss function and AdamW as the optimization algorithm [20]. The choice of cosine annealing allows gradual reduction in the learning rate, which helps avoid suboptimal local minima. AdamW, in turn, provides better generalization than standard Adam due to its decoupled weight decay mechanism.

### 2.6. RETFound Model

The RETFound architecture is based on a masked autoencoder (MAE) paradigm, where an image is partially masked and the network learns to reconstruct the masked patches [16]. For the color fundus image modality, a mask ratio of 0.75 was used. The encoder is a ViT-Large composed of 24 transformer blocks (each with multi-head self-attention and MLP layers), processing patch embeddings of size 1024. The decoder is a lighter ViT with 8 transformer blocks and embedding dimension 512; it takes the encoded features plus inserted “masked token” placeholders and reconstructs the removed patches via linear projection to pixel space. In training, the MAE was pretrained self-supervised on 904,170 fundus images via reconstruction loss, with a batch size of 1792 (across 8 A100 GPUs), over 800 epochs. The final pretrained checkpoint of RETFound is used for downstream adaptation [16].

During adaptation for our classification task, we discarded the decoder and used only the encoder to extract features. At the end of the encoder, we added a global average pooling layer and a dense layer of 4 neurons (the number of target classes). This layer had a softmax activation function [16]. We fine-tuned the RETFound model for 100 epochs with early stopping using a batch size of 16 images and a linear warmup with cosine annealing learning rate schedule. We also used the weighted categorical cross entropy as the loss function and AdamW as the optimization algorithm [20]. The use of SSL makes RETFound highly data-efficient and capable of adapting well to new tasks with minimal labeled data, an important feature in medical domains.

### 2.7. Ensemble Model

The final model was built by stacking the individual models and including a meta-learner at the end. For this task, the outputs of the ResNetRS and RETFound models were concatenated to yield a vector of 8 elements. This vector was the input of the meta-learner. The meta-learner included a dense layer of 6 neurons with ReLU and a dense layer of 4 neurons with softmax at the output.

The ensemble model was also trained for 100 epochs with early stopping using a batch size of 16 images and a linear warmup with cosine annealing learning rate schedule. In this stage, we used the focal loss function to address class imbalance [21]. Focal loss is particularly effective in situations where certain AMD classes are underrepresented, as it reduces the weight of well-classified samples and focuses learning on harder examples.

## 3. Results

Results for the individual models and the ensemble model were obtained in terms of accuracy (ACC), sensitivity (SE), precision (PR), F1-score and quadratic weighted kappa (QWK) using the test set of the AREDS dataset (15,003 images). The ensemble model was also evaluated on the test set of the private dataset (420 images). As shown in Table 1, the ResNetRS model surpasses the RETFound model, and the ensemble model performs better than both the individual ones. These results confirm that the ensemble strategy successfully integrates the strengths of the individual networks, leading to a more effective classification.

Although the ensemble model achieved only a modest improvement in accuracy compared to ResNetRS (+0.86%), it produced a higher QWK value (+0.008), which is particularly meaningful for ordinal classification tasks such as AMD grading. Moreover, the ensemble reduced variability between classes, increasing sensitivity for Intermediate and Late AMD while maintaining high precision. These results confirm that the ensemble provides complementary information that enhances the model’s clinical reliability beyond simple accuracy gains.

**Figure 2:**
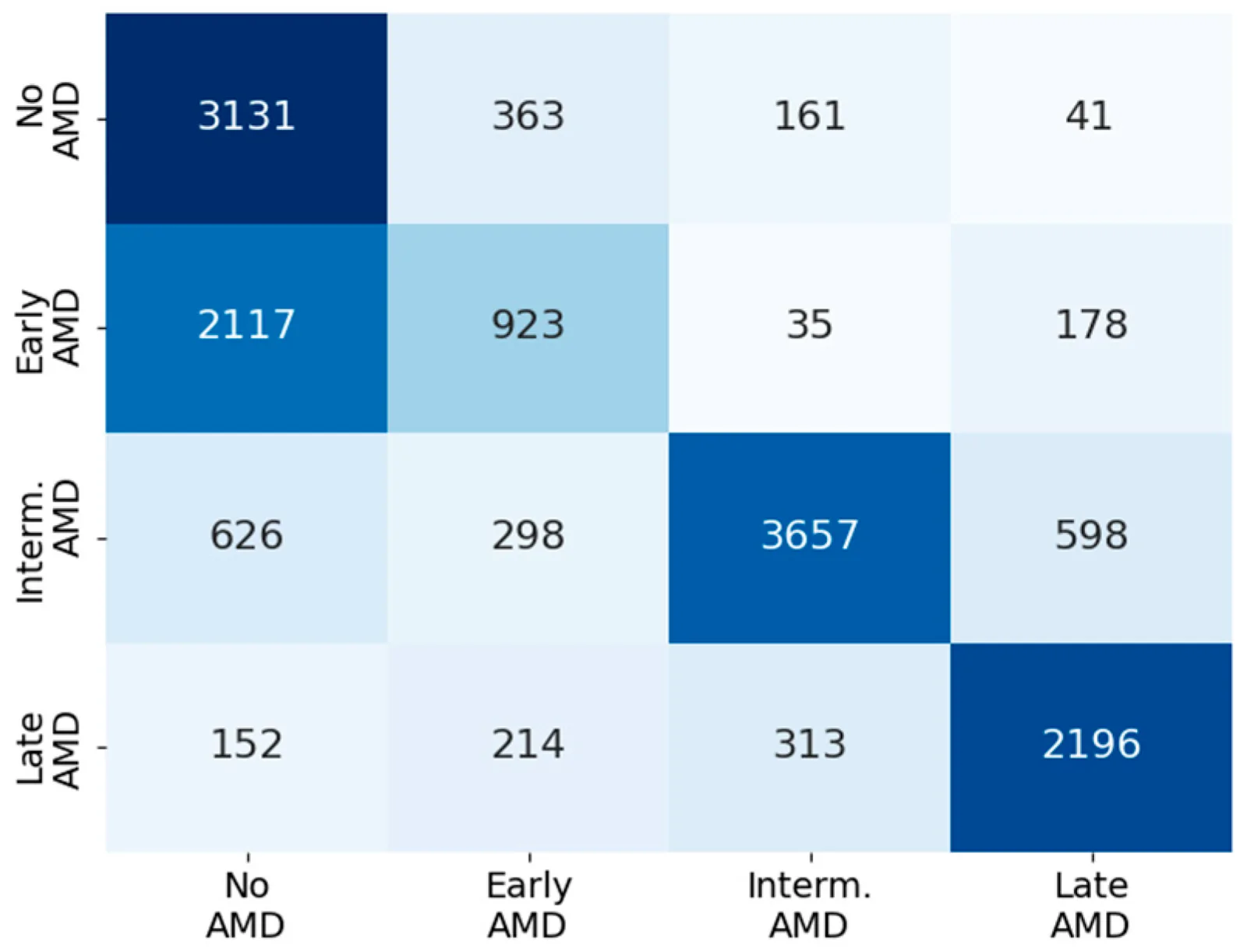
Confusion matrix for the test set of the AREDS dataset using the proposed ensemble model.

In Figure 2, we show the confusion matrix for the test set of AREDS using the proposed ensemble model. As observed, the class “Early AMD” is frequently misclassified as “No AMD”. This misclassification likely results from the subtle visual differences between early AMD and the healthy retina, which can be challenging even for human experts to detect. Drusen, for example, may be small and sparse in early stages, making them less visible depending on image quality or lighting. The other 3 classes are classified with a notably better performance, as visually suggested by the darker diagonal entries in the confusion matrix for those categories.

To further understand the performance distribution, we analyzed the per-class metrics, as shown in Table 2. The ensemble model achieved the following SE values on the AREDS test set: 84.71% for ‘No AMD’, 28.37% for ‘Early AMD’, 70.61% for ‘Intermediate AMD’, and 76.38% for ‘Late AMD’. The particularly low sensitivity in the ‘Early AMD’ category confirms the confusion shown in the confusion matrix. On the other hand, the high sensitivity for ‘No AMD’ and ‘Late AMD’ supports the model’s capacity to distinguish between the extremes of the disease spectrum.

PR values followed a similar pattern (Table 2): the model was most precise in identifying ‘Late AMD’ and least precise in ‘Early AMD’. These results suggest that the ensemble approach is particularly well-suited for recognizing clear pathological changes but less reliable for subtle indicators of disease onset.

Notably, the ensemble model achieved its highest overall performance when evaluated on the private dataset, reaching a QWK of 0.8046. This may be attributed to better image quality, more consistent labeling, or reduced class imbalance. The performance boost on this independent set supports the model’s generalizability, which is crucial for real-world deployment.

## 4. Discussion

In this work, we have developed an ensemble model for fundus image analysis to aid in AMD grading. Our approach combines 2 individual models of a very different nature: (1) a CNN with ResNetRS architecture based on SL, and (2) a network with ViT architecture based on SSL (RETFound). To the best of our knowledge, this is the first time that RETFound has been combined with a CNN model in an ensemble fashion for AMD grading.

The decision to implement a hybrid ensemble was based on empirical observations showing that standard pre-trained models, although effective individually, exhibited different error patterns. CNNs such as ResNetRS captured detailed drusen and pigment changes, whereas RETFound focused on broader retinal structures. By combining their outputs through a meta-learner, the ensemble effectively merged local and global feature representations, leading to improved consistency across datasets. This supports the theoretical rationale for hybrid modeling in retinal image analysis.

For our experiments, we used the largest dataset of fundus images publicly available in the context of AMD. Additionally, our approach was tested on an independent private set. This comprehensive evaluation provides insights into both the model’s internal validity and its external generalizability.

Results in Table 1 show that the ensemble model outperforms the individual models in almost every metric, achieving ACC = 66.03%, SE = 66.03%, PR = 68.20%, F1-score = 0.6510 and QWK = 0.7364. The incremental quantitative gains of the ensemble should be interpreted in the context of their qualitative impact. The hybrid model achieved more stable predictions across disease stages, showing fewer extreme misclassifications (e.g., Early vs. Late AMD) and improved consistency between datasets. As we hypothesized, the combination of ResNetRS and RETFound allowed us to reduce the prediction error and improve the model generalization. The results obtained using the private dataset were also consistent, with ACC = 70.95%, SE = 70.95%, PR = 66.71%, F1-score = 0.6651 and QWK = 0.8046. These results show an effective adaptation of the proposed method to independent datasets. It is worth noting that the ensemble was not merely averaging predictions but rather learning from the outputs of both models through a dedicated meta-learner, which enhances the decision process.

When comparing the individual models with each other, the ResNetRS model shows a higher performance than the RETFound model. Despite the promising results of the recent approach ViT-SSL, this work supports that a modern CNN architecture, such as ResNetRS, together with transfer learning can achieve a superior performance for AMD classification. Nonetheless, the RETFound model still contributes meaningful representations to the ensemble, especially in ambiguous cases where spatial attention becomes crucial.

The improvement observed in the private dataset is particularly promising. In real-world clinical deployments, models are often exposed to data distributions that differ significantly from those used in training. Thus, a method that generalizes well across centers, devices, and patient demographics is highly desirable. Our findings suggest that the ensemble model is capable of transferring knowledge effectively between domains.

Our results can be compared to those obtained in the literature for AMD grading, as shown in Table 3. However, the comparisons should be made with caution since the number of target classes and the specific datasets are different among studies. In the work of Burlina et al. [6], a high ACC = 83.20% was obtained, but QWK reached 0.6540, which is lower than our QWK = 0.7364. Like our study, they used the AREDS dataset and considered 4 AMD degrees. However, the provided results were computed applying a 5-fold cross validation over a reduced subset of 67,401 images. Peng et al. also used the AREDS dataset [14]. Nonetheless, they selected 38,884 images for testing and classified AMD into 6 severity levels. Their ACC = 67.10% performed similarly to ours (ACC = 66.03%), while their QWK = 0.5580 was considerably lower than the one obtained with our approach (QWK = 0.7364). At the end, QWK is the most representative metric for multiclass classification [22]. Our higher QWK indicates that the predictions of our model more closely align with the ordinal structure of the ground truth labels, making the system more trustworthy in clinical applications. Although direct comparisons cannot be made, we can conclude, in general terms, that our ensemble model achieves a higher performance than the previous studies.

Although the proposed ensemble achieved an overall accuracy of 66.03% and a QWK of 0.7364, these results should be interpreted in the context of AMD screening rather than as a final diagnostic decision. In clinical terms, a QWK between 0.61 and 0.80 is considered indicative of substantial agreement with expert grading [24], suggesting that the model can reliably support ophthalmologists in large-scale screening programs. Most misclassifications occurred between “No AMD” and “Early AMD,” categories that are often difficult to distinguish even for human experts and have limited therapeutic implications. Conversely, the model showed strong performance for the detection of “Intermediate” and “Late AMD,” which are the stages requiring closer clinical follow-up or treatment. This behavior indicates that potential risks from misclassification are minimal from a clinical management standpoint.

Regarding computational complexity, the ensemble combines ResNetRS (≈200 k parameters) and RETFound (≈300 k parameters), for a total of nearly 500 k parameters. After both base models are trained and frozen, only the meta-learner remains trainable, consisting of 82 parameters. The ensemble was trained on an NVIDIA GeForce RTX 4080 GPU (batch size = 16) with an average training time of about 21 min per epoch. Once deployed, the model performs inference in under one second per image, confirming its efficiency for large-scale screening scenarios. These results show that the proposed hybrid ensemble achieves high accuracy without imposing substantial computational demands, supporting its practicality for research and potential clinical use.

The present work has limitations that should be mentioned. First, the images from AREDS were collected between 1992 and 2004 using the cameras available in that moment. Therefore, they show overall lower quality and resolution than images obtained with modern cameras. This is the reason why our validation procedure required a fine-tuning stage using part of the private dataset. In the future, it would be desirable to improve the robustness of the model for modern fundus images and avoid the need for adjustments. Moreover, the datasets used in this study—although representative—may not fully reflect the diversity of acquisition protocols, ethnic backgrounds, or disease manifestations found in real-world clinical settings. External validation on larger, multi-center datasets will be essential to confirm the generalizability of the proposed approach. Second, the model showed relatively low sensitivity for the detection of Early AMD, which represents an important clinical limitation since early identification is critical for effective prevention and monitoring. This reduced sensitivity is likely related to the subtle and often ambiguous visual signs of early disease, which are difficult to distinguish even for expert graders. Future work should focus on improving the detection of early-stage features by incorporating higher-resolution data, targeted augmentation, or attention mechanisms. Third, although the present study demonstrates the potential of the proposed hybrid ensemble, we recognize that a comprehensive ablation study comparing multiple ensemble strategies and recent hybrid architectures would provide further insight. Future research will address this aspect in detail, exploring how different combinations of CNN and transformer-based models, as well as alternative fusion strategies, affect performance and interpretability. Ablation studies would allow us to determine an optimized combination of the individual models. Fourth, the computational cost of the ensemble was only partially characterized. While preliminary figures were reported, a complete benchmarking of computational metrics would be valuable to evaluate the trade-offs between model complexity and accuracy. Finally, the present study lacks interpretability in the predictions. The use of explainable artificial intelligence (XAI) would help better comprehend the contribution of the individual models to the ensemble. In addition, a XAI analysis would increase the confidence in the model in a clinical setting. Future work may integrate error analysis and techniques such as Grad-CAM or attention rollout to highlight the retinal regions that influence predictions, thus promoting clinical interpretability. Moreover, the current study did not include a prospective assessment of the model’s real-world impact in terms of clinical workflow or workload reduction. Such evaluations, combined with XAI visualizations, will be necessary to ensure that the system can be safely and effectively integrated into ophthalmic practice.

## 5. Conclusions

The proposed ensemble strategy for AMD grading combines 2 individual models built on completely different foundations. The first model was based on a ResNetRS architecture and SL (pretrained in a supervised way on a general-purpose image dataset). The second model, RETFound, was based on a ViT architecture and SSL (pretrained in a self-supervised way using retinal fundus images). This is the first study to combine RETFound and a CNN model in an ensemble architecture for AMD grading.

Individually, the first model surpassed the second one. However, they proved to provide complementary information for the classification task. The success of this combination demonstrates the value of hybrid modeling in medical image analysis, especially when dealing with heterogeneous and imbalanced data.

The proposed ensemble outperformed the previous approaches in the literature on AREDS, the largest fundus image database for AMD research. The method was also validated using a private database. The strategy presented in this study could potentially contribute to improving the screening of AMD in a clinical setting. An early diagnosis would allow for adequate treatment and better eye care. Moreover, such an automated system could help reduce the workload of specialists and the associated costs by assisting in preliminary image screening. In addition, the general framework of our approach—combining CNNs with ViT-based foundation models—could be extended to other ophthalmological conditions such as diabetic retinopathy or glaucoma, opening new avenues for future research and clinical deployment.

## Figures and Tables

**Table 1:** Results of the proposed method on the test sets of the AREDS and private datasets.

Model	Dataset	ACC	SE	PR	F1-Score	QWK
ResNetRS	AREDS	65.17	65.17	68.35	0.6580	0.7284
RETFound	AREDS	57.65	57.65	60.24	0.5576	0.6911
Ensemble	AREDS	66.03	66.03	68.20	0.6510	0.7364
Ensemble	Private	70.95	70.95	66.71	0.6651	0.8046

**Table 2:** Per-class performance metrics on the test set of the AREDS dataset.

Class	SE (%)	PR (%)	F1-Score
No AMD	84.71	51.96	0.6441
Early AMD	28.37	51.33	0.3655
Intermediate AMD	70.61	87.78	0.7827
Late AMD	76.38	72.88	0.7459

**Table 3:** Comparison with previous studies.

Study	Dataset	ACC	QWK
Phan et al. (2016) [23]	Private	62.70	-
Burlina et al. (2017) [6]	AREDS	83.20	0.6540
Peng et al. (2019) [14]	AREDS	67.10	0.5580
Proposed method	AREDS	66.03	0.7364
Proposed method	Private	70.95	0.8046

## Data Availability

The data presented in this study are openly available in the National Eye Institute (NEI) Age-Related Eye Disease Study (AREDS) at https://www.ncbi.nlm.nih.gov/projects/gap/cgi-bin/study.cgi?study_id=phs000001.v3.p1 (accessed on 15 October 2025), reference number phs000001.v3.p1.
